# Supplementary material for: Content-rich biological network constructed by mining PubMed abstracts
Source: BMC Bioinformatics. 2004 Oct 8;5:147. doi: 10.1186/1471-2105-5-147 (PMC528731; doi:10.1186/1471-2105-5-147)
Supplement: Additional File 5 — The original Chilibot query results of the term "long-term potentiation (LTP)" and 22 other terms, limiting the latest references analyzed to the years 1990, 1995, 2000, and 2004. [file 1471-2105-5-147-S5.bz2 › chilibotAdditionalFile5/ltp2000/html/TRKB_NMDA.html]

 


 **TRKB** and **NMDA** 
  
Found 25 abstracts in PubMed,  **25 abstracts were retrieved and analyzed**.  


---

 Search Google  |
 PDF files only 
|  EDU domain only 

---

**Interactive relationship** (e.g. stimulation, inhibition, etc)

- We suggest that  **NMDA**  activates the  **TrkB**  receptor via a BDNF autocrine loop, resulting in neuronal survival.  Ref: 9792641 J Biol Chem, 1998
- Results show that  **trkB**  is strongly induced in these neurons in an N methyl D aspartate  **NMDA**  receptor dependent manner.  Ref: 9191102 Brain Res Mol Brain Res, 1997
- The increase in BDNF in the medium is followed by enhanced  **TrkB**  tyrosine phosphorylation, suggesting that  **NMDA**  increases the release of BDNF and therefore the activity of  **TrkB**  receptors.  Ref: 9792641 J Biol Chem, 1998

- :-)

  **Parallel relationship** (e.g. studied together, co-existance, homology, etc.)

  - These findings suggest that BDNF increases glutamate release and N methyl D aspartate  **NMDA**  channel gated calcium influx via  **TrkB**  and regulates the frequency and the amplitude of calcium oscillations.  Ref: 9459549 Brain Res, 1997
  - Both compounds blocked the  **NMDA**  mediated  **TrkB**  tyrosine phosphorylation and subsequently its neuroprotective properties.  Ref: 9792641 J Biol Chem, 1998
  - This decrease is overcome by anti BDNF or  **TrkB**  IgG blocking reagents or by CNQX, a non  **NMDA**  glutamate receptor antagonist.  Ref: 9570788 J Neurosci, 1998
  - The results indicate that acute exposure to BDNF increases  **NMDA**  single channel open probability via postsynaptic  **trkB**  receptors.  Ref: 11054804 J Neurosci Res, 2000
  - The noncompetitive N methyl D aspartate  **NMDA**  antagonist ketamine blocked NGF, BDNF, and  **trkB**  mRNA increases in the hippocampus and cerebral cortex, but not in the amygdala and piriform cortex.  Ref: 8635431 Epilepsia, 1996
  - To examine whether BDNF and  **TrkB**  signaling play a role in the  **NMDA**  mediated neuroprotective properties, neurons were exposed to soluble  **trkB**  receptor IgG fusion protein, which is known to inhibit the activity of extracellular BDNF, and to K252a, a tyrosine kinase inhibitor.  Ref: 9792641 J Biol Chem, 1998
